# "Brace Technology" Thematic Series - The ScoliOlogiC^® ^Chêneau light™ brace in the treatment of scoliosis

**DOI:** 10.1186/1748-7161-5-19

**Published:** 2010-09-06

**Authors:** Hans-Rudolf Weiss, Mario Werkmann

**Affiliations:** 1 Orthopedic Rehabilitation Services, D-55457 Gensingen, Alzeyerstr. 23, Germany; 2 Orthomed Scoliocare, Orthopedic Technical Services, D-55457 Gensingen, Alzeyerstr. 23, Germany

## Abstract

**Background:**

Bracing concepts in use today for the treatment of scoliosis include symmetric and asymmetric hard braces usually made of polyethylene (PE) and soft braces. The plaster cast method worldwide seems to be the most practiced technique for the construction of hard braces at the moment. CAD (Computer Aided Design) systems are available which allow brace adjustments without plaster. Another possibility is the use of the ScoliOlogiC™ off the shelf system enabling the Certified Prosthetist and Orthotist (CPO) to construct a light brace for scoliosis correction from a variety of pattern specific shells to be connected to an anterior and a posterior upright. This Chêneau light™ brace, developed according to the Chêneau principles, promises a reduced impediment of quality of life in the brace. The correction effects of the first 81 patients (main diagnosis Adolescent Idiopathic Scoliosis (AIS) [n = 64] or Early Onset Scoliosis (EOS) [n = 15]), treated according to the principles of the Chêneau light™ brace have shown a satisfactory in-brace correction exceeding 50% of the initial Cobb angle.

**Brace description:**

The ScoliOlogiC^® ^off the shelf bracing system enables the CPO to construct a light brace for scoliosis correction from a variety of pattern specific shells to be connected to an anterior and a posterior upright. This brace, when finally adjusted is called Chêneau light™ brace. The advantage of this new bracing system is that the brace is available immediately, is easily adjustable and that it can also be easily modified. This avoids construction periods of sometimes more than 6 weeks, where the curve may drastically increase during periods of fast growth. The disadvantage of this bracing system is that there is a wide variability of possibilities to arrange the different shells during adjustment.

**Results:**

The Cobb angle in the whole group was reduced by an average of 16,4°, which corresponds to a correction effect of 51%. The differences were highly significant in the T-test (T = 17,4; p < 0,001). The best correction effects achieved with Chêneau braces reported in literature so far are about 40% in two different studies. The correction effect was highest in lumbar and thoracolumbar curve patterns (62%; n = 18). In thoracic scoliosis the correction effect was 36% (n = 41) and in double major curve patterns 50% (n = 22). The correction effect was affected in a slightly negative way due to age (r = -0,24; p = 0,014), negatively with the Risser stage (-0,29; p = 0,0096) and correlated negatively with the Cobb angle measured before treatment (r = -0,43; p < 0,0001).

**Conclusions:**

The use of the Chêneau light™ brace leads to correction effects above average when compared to correction effects of other braces described in literature. The reduction of material seems to increase patient's comfort and reduces the stress patients may suffer from whilst in the brace.

80% of the adolescent population of scoliosis patients can be braced with the Chêneau light™ brace. In certain patterns of curvature and in the younger population with an age of less than 11 years, other approaches have to be used, such as plaster based bracing or the application of CAD/CAM based orthoses.

## Introduction

Bracing concepts in use today for the treatment of scoliosis include symmetric and asymmetric hard braces usually made of PE and soft braces. The latest developments in the field of bracing, aim at improving specificity (1) and at a proper sagittal realignment (2).

The plaster cast method worldwide seems to be the most practiced technique for the construction of hard braces at the moment. CAD (Computer Aided Design) systems are available, which allow brace adjustments without plaster. Another development however, is the ScoliOlogiC™ off the shelf system enabling the CPO to construct a light brace for scoliosis correction from a variety of pattern specific shells to be connected to an anterior and a posterior upright [1]. This Chêneau light™ brace, constructed according to the Chêneau principles, promises a reduced impediment of quality of life in the brace. The correction effects of the first 81 patients (main diagnosis Adolescent Idiopathic Scoliosis (AIS) [n = 64] or Early Onset Scoliosis (EOS) [n = 15]), treated according to the principles of the Chêneau light™ brace have shown a satisfactory in-brace correction exceeding 50% of the initial Cobb angle [2].

**Figure 1:**
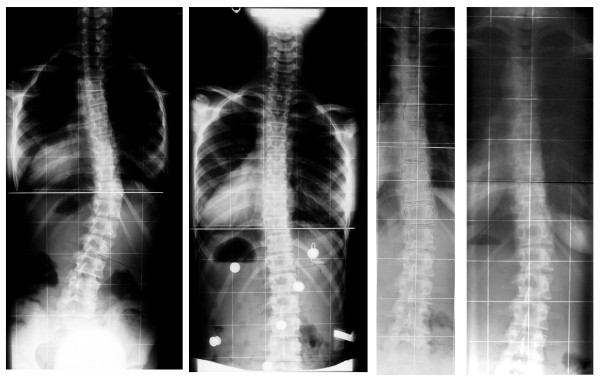
**Overcorrection of a curve coming from 37° to -16° in a custom plaster based Chêneau brace from the 90's**. In this case, not only progression has been stopped. At weaning off the curve is at 14°, two years after weaning off - final result: 16°. No further treatment necessary. (Weiss 2010).

**Figure 2:**
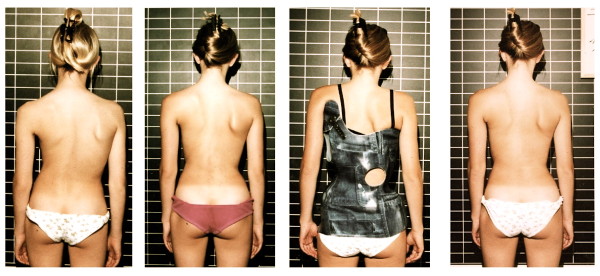
**Clinical follow-up with a significant improvement two years after weaning off**. On the *left *initially a decompensated right thoracic scoliosis is visible, two years after weaning off (*right*) the trunk is more balanced and the patient copes well with scoliosis. In between the first Chêneau brace of the patient (Standard 1997) and one intermediate result is visible.

Although the effect of brace treatment has been questioned, [3] there is evidence that brace treatment can stop curvature progression [4-9] (Fig.1.), reduce the frequency of surgery [10-12] and improve cosmetic appearance [13-15] (Fig. 2.). Poor cosmetic appearance for the patient may be the most important problem, which can be solved or at least reduced by the use of advanced bracing techniques including the best possible correction principles available to date [13].

## History of the Chêneau light™ brace

The Chêneau light™ brace in principle is a Chêneau derivative. The Chêneau brace was developed before 1978 [16]. As the first developments were made in Münster, Germany, the brace was initially called CTM-brace (Chêneau-Toulouse-Münster). Jacques Chêneau, who used to live in Toulouse, spent a few years in Münster, where he braced patients at the orthopedic department of the university there. In 1985 the first end-result study was published with in brace correction effects of more than 40% of the initial value [7] and final result superior to the end-results of the Milwaukee study from the same centre [17]. The initial Chêneau brace was upgraded in 1995 and from this year on, a new version each year was promoted by the inventor during the courses organized in Germany together with Dr. Weiss in Bad Sobernheim and Prof. Neff in Berlin. A working relationship between Dr. Chêneau, Dr. Weiss and Dr. Rigo began in Bad Sobernheim, Germany towards the end of the 90's, which resulted in a collaboration in publishing a book presenting the 1999 standard of the Chêneau brace [18].

At the beginning of the new century Dr. Chêneau was working on the first CAD/CAM system supported by a company called IPOS in Germany.

Other CAD/CAM systems developed in Germany applying the Chêneau principles, such as the Regnier system and the RSC-brace. The latter was developed by Dr. Rigo and was finally improved to be ready for marketing with the help of Dr. Weiss. While Dr. Rigo in Barcelona was producing plaster cast positives of as many curve patterns as possible for digitalization, in order to increase the library, Dr. Weiss was testing the new modules and advised the company on how to improve the individual pattern specific modules.

The first ideas to produce a pattern specific off the shelf bracing system in 2002 were initially overlayed by the oncoming development of the pattern specific CAD/CAM Chêneau braces. In 2005 however, the patent application was written and the first hand-made Chêneau light braces were applied. In the summer of 2006 the fabrication of the ScoliOlogiC^® ^off the shelf bracing system for the adjustment of Chêneau light™ braces began.

## Theoretical principles

Aim of this new development was to make the brace lighter, finer, easier to wear, and by this, allowing a better quality of life for the patients with scoliosis under brace treatment.

**Figure 3:**
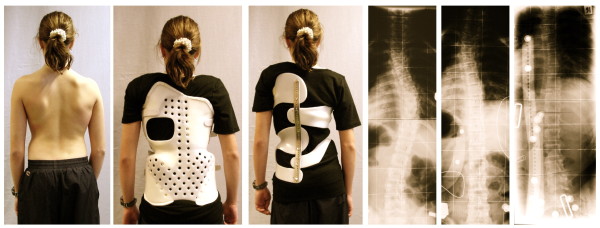
**13-year old girl with AIS (39° thoracic)**. In the previous brace she had 22° high thoracic, 12° low thoracic and 5° lumbar, while in the Chêneau light^® ^brace she has 22° high thoracic, 8° low thoracic and 11° lumbar. The lumbar correction has not been improved after this x-ray in order to achieve a better balance of curves after treatment and a better cosmetic result. The reduction of material in the Chêneau light^® ^brace compared to the previous brace is clearly visible. Brace change was necessary due to severe pains in the previous brace [2].

This is accomplished by using less material in comparison to traditional bracing systems, intended for scoliosis treatment (Fig. 3).

**Figure 4:**
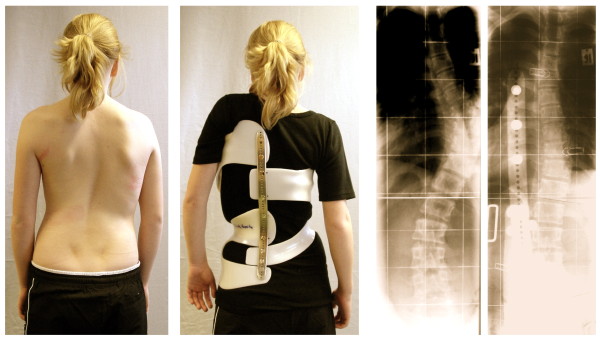
**13-year old girl with 50° Cobb corrected to 16° in the brace**. This is only possible when the brace is adjusted well and the voids (in this case a void ventral on the rib hump side and a void lateral to the concavity) are clearly visible. In order to achieve maximum possible 3D-correction the concavity must be opened in order to allow a corrective rib movement.

Many 3-point pressure systems are applied on the frontal, coronal and sagittal plane. Opposite to every pressure area an expansion void is implemented. This enables the desired corrective movement (Fig. 4.) and - when adjusted properly - avoids compression effects leading to pressure sores. As a matter of fact in today's Chêneau braces pressure sores have become a very rare complication.

The brace action (Patient shown on fig. 4 and two other examples) is demonstrated on a morphing video, which can be seen on this link: http://www.youtube.com/watch?v=peHWmtdRorU

Pattern specific bracing is desirable to allow, to correct the individual curve patterns appropriately, as theoretically there might be an unlimited number of curve patterns. Therefore, a classification is necessary to come as close as possible to address the biomechanical properties of the individual curve pattern of the patient treated.

After the first curve patterns were identified by Ponseti and Friedmann [19], and Moe and Kettleson [20] for surgical means, in the late 70's a simple functional classification for approaching different curve patterns with the help of physiotherapy was established by Lehnert-Schroth [21,22]. This classification simply distinguished between so called 3- and 4-curve patterns.

Chêneau also used this simple classification for the construction of his braces.

The King classification [23] distinguished between 5 different (thoracic) curve patterns and was established in the 80's to help the surgeons to approach the curves properly during operations.

The Lenke classification [24], which is rather complex was developed by surgeons, because the use of King Classification had lead to imbalanced post surgical results and seemed to lack reliability.

It was Rigo [25] who implemented a new classification for brace treatment with 15 different curve patterns, derived from the Lenke classification [24]. All those curve patterns demand individual principles of correction in 3 D, however, 5 key patterns have been identified which we can start working with in everyday practice [26].

This year Rigo came up with a brand new classification [27], which, in our opinion is still not simple enough to be used by CPOs generally.

**Figure 5:**
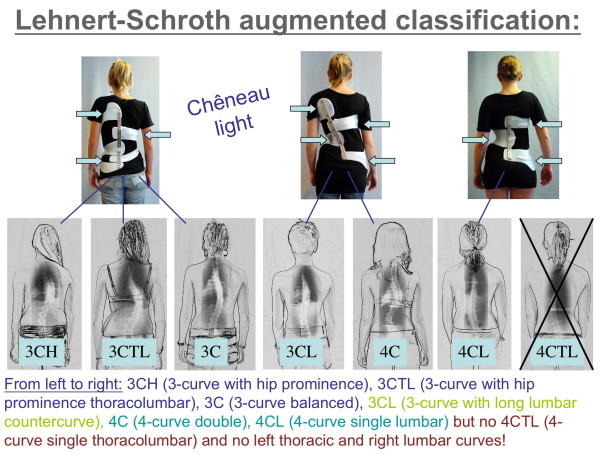
**The augmented classification according to Lehnert-Schroth**. Curvatures decompensated to the thoracic convex side have to be regarded as functional 3-curve type curvatures, when balanced or decompensated to the thoracic concave side (lumbar curves as big as thoracic ones or even bigger) per definition they are functional 4-curve types. As can be seen, the 3-curve lumbar is a 3-curve pattern, but treated like 4-curve with reduced correction in the lumbar curve.

Therefore, the first author is now returning to the simple functional classification by Lehnert-Schroth. The principal subdivision of functional 3 and functional 4 curves still seems to work for physiotherapy and can easily be augmented to the needs of the CPO. This augmented Lehnert-Schroth classification [28] can be seen on Fig. 5 as well as the braces used to address the individual curve patterns.

## Brace description

The ScoliOlogiC^® ^off the shelf bracing system enables the CPO to construct a light brace for scoliosis correction from a variety of pattern specific shells to be connected to an anterior and a posterior upright. This brace is called Chêneau light™ brace. The advantage of this new bracing system is that the brace is available immediately, easily adjustable and that it can also be easily modified. This avoids construction periods of sometimes more than 6 weeks, where the curve may drastically increase during periods of fast growth. The disadvantage of this bracing system is that there is a wide variability of possibilities to arrange the different shells during adjustment. Therefore the technician has to acquire a deep understanding of basic biomechanics, functional diagnosis and curve pattern identification before being able to apply „Chêneau light" braces.

Shells are available for the treatment of right thoracic and left lumbar curves in three sizes allowing brace adjustments for most of the adolescent patients. For patients with thoracolumbar curve patterns, for left thoracic, right lumbar curve patterns and for smaller sizes a Chêneau light™ brace can be constructed using the plaster cast technique.

**Figure 6:**
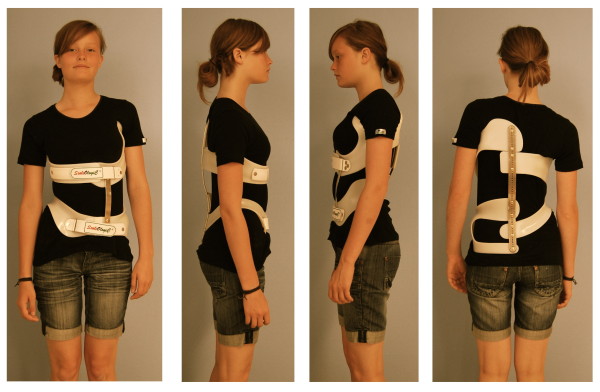
**A brace to address the functional 3-curve patterns for right thoracic curves from all four sides**. The static overcorrection to the concave side is already visible in this "try-on" brace not yet cut and finalized. The dorsal upright is bent physiologically which can be seen from the side. A final 3-curve brace can be seen on Fig. 4. There are no shells available for left thoracic and right lumbar curvatures.

**Figure 7:**
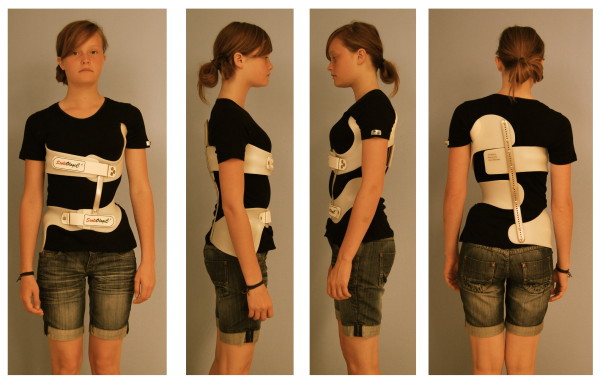
**A brace to address functional 4-curve patterns for right thoracic and left lumbar curves from all four sides**. The static re-compensation of the trunk segments is already visible in this "try-on" brace not yet cut and finalized. The dorsal upright is bent physiologically which can be seen from the side. A functional 4-curve pattern brace in its final form can be seen on Fig. 13. There are no shells available for left thoracic and right lumbar curvatures.

**Figure 8:**
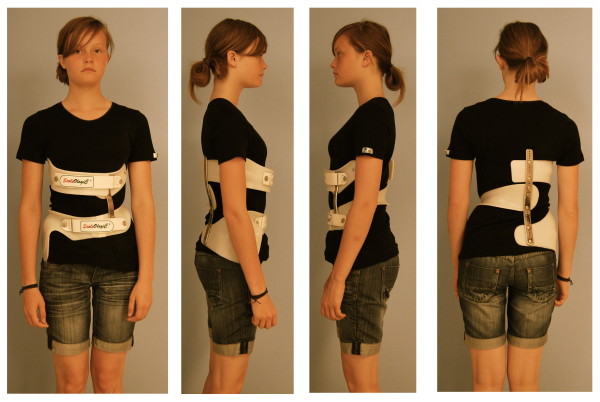
**A short brace cut from a functional 4-curve pattern brace**.

Braces to address functional 3-curve patterns (Fig. 6) and braces to address functional 4-curve patterns (Fig. 7) are available. In single lumbar curves the 4-curve brace is applied and the upper shell, carrying the axillary pressure area is cut, as is the dorsal upright (Fig. 8).

The brace is usually assembled as a standard "try on" brace first using the drill holes marked on the individual shells. After that the brace is adjusted according to the individual curve pattern with the help of the pattern specific blueprints (Additional file 1.).

## Practical issues

### How to prescribe the brace

The Chêneau light™ brace is not prescribed per se. The prescription does not contain the brace name, but a Chêneau brace is prescribed and the curve pattern of the individual patient is submitted as well. The Cobb angles of all curvatures should also be visible on the prescription

Additionally, a construction plan (Additional file 2.) for the brace prescribed is attached to the prescription and should a brace have to be renewed, a further sheet, giving justification (Additional file 3.) as to why a new brace has to be prescribed, should be included.

With this Chêneau prescription, the patient can go to any workshop near home to get his or her brace done using plaster casting, CAD/CAM system braces or Chêneau light braces. If the patient makes the decision to go to the workshop in Gensingen, the CPOs show diverse possible Chêneau derivates so as to enable the patient to decide on the brace type himself. When the Chêneau light™ brace has been chosen the CPO starts the adjustment with the "try-on" brace.

### How to build the brace

**Figure 9:**
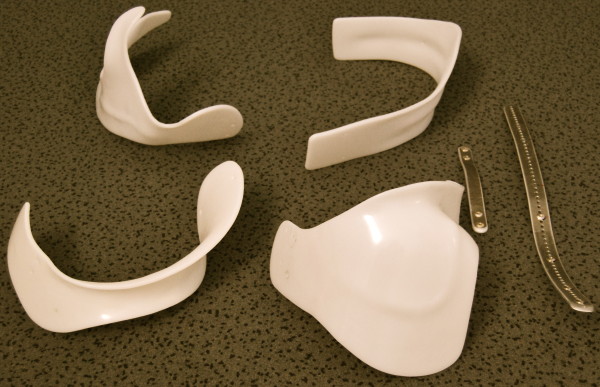
**Chêneau light out of the box!" The individual parts can be seen before they are adjusted for a "try-on" brace**.

The brace consists of four shells, two uprights and the straps with attachments (Fig. 9). First the lumbar shell is attached with rivets (in the "try-on" braces the shells might be attached with screws first) at the lordosis apex of the sagittally pre-bent dorsal upright. The dorsal upright may be more or less inclined in frontal plane depending on the individual curve pattern of the patient treated.

After that the pelvic shell is attached, then the thoracic and finally the axillary shell.

**Figure 10:**
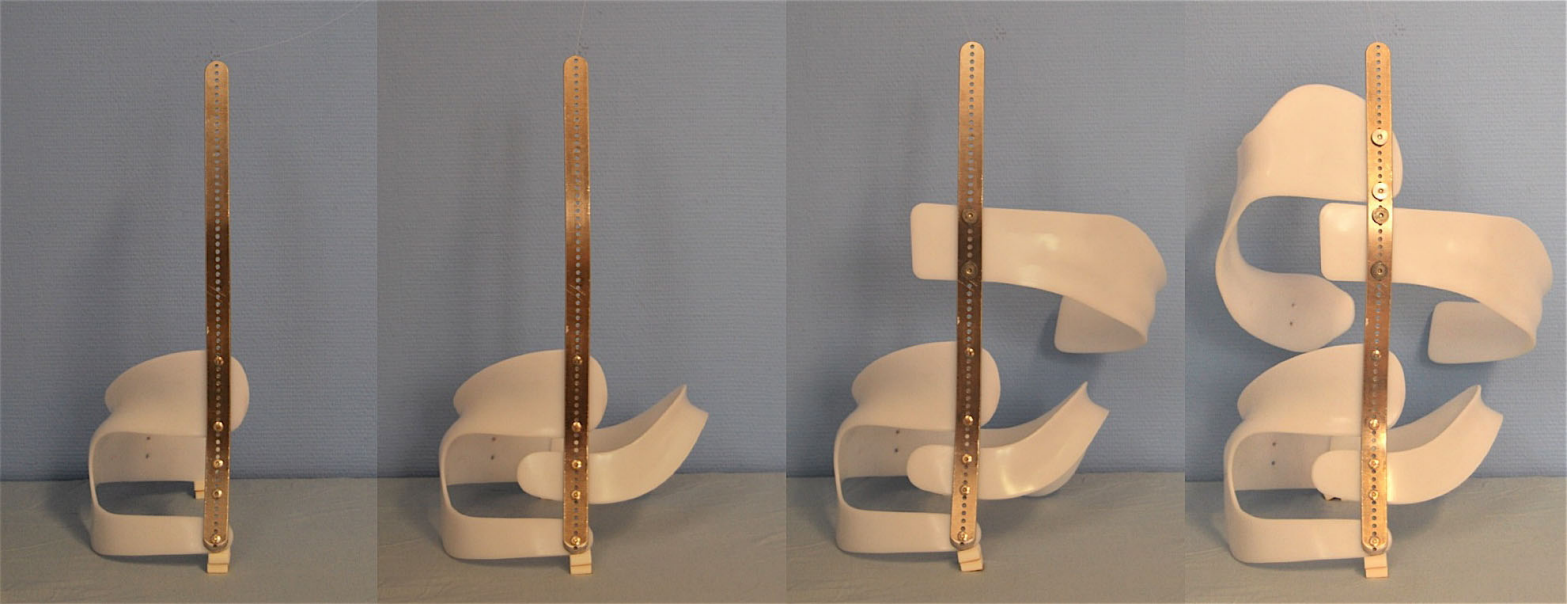
**Step by step construction of a "try-on" brace**. First the lumbar shell is attached with rivets (in the "try-on" braces the shells might be attached with screws first) at the lordosis apex of the pre-bent dorsal upright. After that the pelvic shell is attached, then the thoracic and finally the axillary shell. Because the individual shells might be tilted, the strap attachment is fitted finally after the shells are in their final position before the shell edges are cut in order to finalize and minimize the brace.

Because the individual shells might be tilted, the strap attachment is the final step before the shell edges are cut in order to finalize and minimize the brace. The step-by-step construction can be seen in Fig. 10. A visual impression on how the brace is adjusted can be found on the following videos: http://www.youtube.com/watch?v=g6Qk7wKEzuI and on the second part of this video:

http://www.youtube.com/watch?v=0P9eKW0Fpis.

### How to check the brace

**Figure 11:**
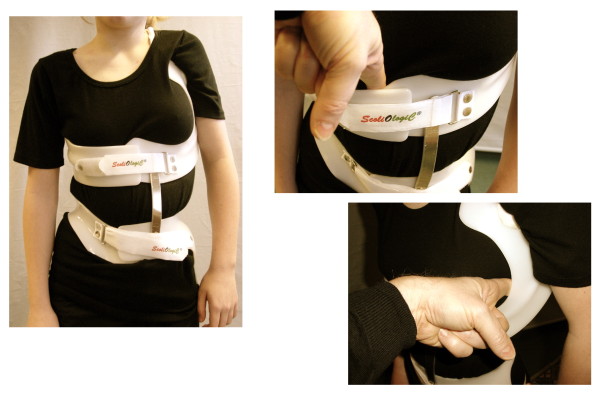
**Controlling the voids**. Ventrally to the rib hump a void is designated in order to allow a corrective movement of the trunk. On the concave side a void is designated as well for the correction in the frontal plane. The voids are tested with the fingers as can be seen on this Fig.

The brace is checked in a standardized way. First of all a verification of the pattern specificity is necessary, after that the shell attachment is checked clinically for the right height in relation to the neighboring shells. Then the voids are controlled (Fig. 11), the adjustment of the uprights, the tilt angles of the shells and the impact the construction clinically has on the patient. This is done by the CPO first and is documented on the „Checklist"(Additional file 4.)

After that the CPO presents the patient and the checklist to the physician, who has another checklist (available in German only, Additional file 5.) for the final clinical check-up.

**Figure 12:**
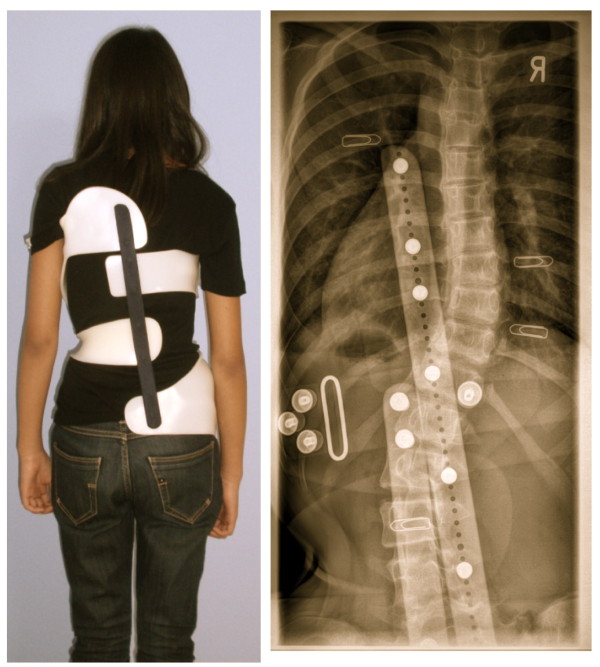
**In-brace x-ray with pad markers visible**. The markers are attached to the apical zones of the pressure areas. In this case an artificial decompensation, caused by the brace is clearly visible. In Double Major curvatures compression effects may arise in the middle of the trunk when the lower ribs are very long inhibiting thoracic realignment, when pushed by the lumbar pad. In this case, the brace has to be reassembled, because the correction effect is not sufficient. Without in-brace x-ray this imbalance would have remained undetected.

After any improvements have been made, the patient is scheduled for the next appointment in 6 weeks in order to have a clinical check-up and the in-brace x-ray completed with pad markers (Fig. 12).

### Protocols

The criteria for bracing are taken one to one from the SOSORT indication guidelines [29].

### Everyday usage

the number of hours per day that the patient will wear the brace in principle is taken one to one from the SOSORT indication guidelines [29].

### Exercises

There are no exercises done in the brace because we aim at maximum possible correction giving no room for further corrections with the help of exercises. However, for the exercises without the brace on, the augmented Lehnert-Schroth classification is also applied http://www.youtube.com/watch?v=eHsCsL7IEaU.

## Results & case reports

At least in Germany the Chêneau brace has been widely reviewed. As early as 1985 the first end-result study was published [7]. The average in-brace correction reported on within this study was 40%. Landauer [6] presented a case series of patients treated with the Chêneau brace with comparable in-brace corrections and comparable end-results.

A prospective controlled study comparing the Chêneau brace with SpineCor has clearly shown the superiority of the Chêneau brace in a sample of patients at actual risk for being progressive, fulfilling the SRS criteria for studies on bracing [9]. After growth only 8% from the SpineCor samples were not progressive and 80% of the Chêneau group. The Cobb angle at treatment begin however, was 21° for the SpineCor sample and 33° for the Chêneau brace sample of patients.

According to Landauer and collaborators [6] two factors are influencing the outcome of brace treatment, both of them being as important as the other: In-brace correction (1) clearly correlates with the final result. The better the in-brace correction, the better the end-result. Compliance (2) is the other important factor. The best possible in-brace correction will not change the prognosis of the patient when the brace is not worn as prescribed.

The in-brace correction in the Chêneau light™ brace in a patient sample with AIS and EOS was satisfying. In-brace corrections exceeding 50% have been reported in literature in a sample of patients with an average Cobb angle of 36° [2].

The Cobb angle in the whole group was reduced by an average of 16,4°, which corresponds to a correction effect of 51%. The differences were highly significant in the T-test (T = 17,4; p < 0,001). The correction effect was highest in lumbar and thoracolumbar curve patterns (62%; n = 18). In thoracic scoliosis the correction effect was 36% (n = 41) and in double major curve patterns 50% (n = 22). The correction effect was affected in a slightly negative way due to age (r = -0,24; p = 0,014), negatively with the Risser stage (-0,29; p = 0,0096) and correlated negatively with the Cobb angle measured before treatment (r = -0,43; p < 0,0001) [2].

In another study a patient sample having had experience with solid hard braces and also with the Chêneau light™ brace, have been evaluated. In the BSSQ^brace^, a questionnaire, designed by Dr. Weiss to test the stress a patient has in a brace, the values for the Chêneau light™ brace were significantly higher, which means the patients had less stress in the Chêneau light™ brace (Score 0 = highest possible stress/score 24 = no stress at all) [30].

The best correction effects achieved with Chêneau braces reported in literature so far are about 40% in two different studies [6,7], therefore the in-brace corrections as achieved with the Chêneau light™ brace are the best in-brace corrections reported for Chêneau braces in international literature.

**Figure 13:**
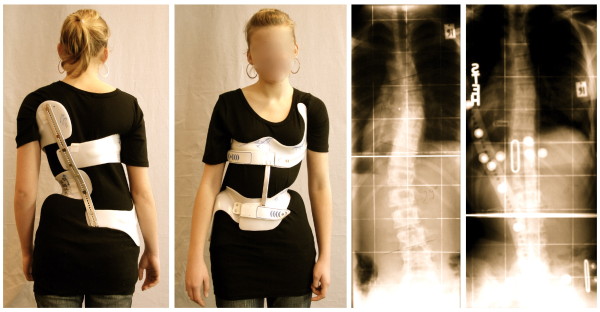
**13-year old girl with double major curvature and with an in-brace correction exceeding 60% in both of the curves**.

**Figure 14:**
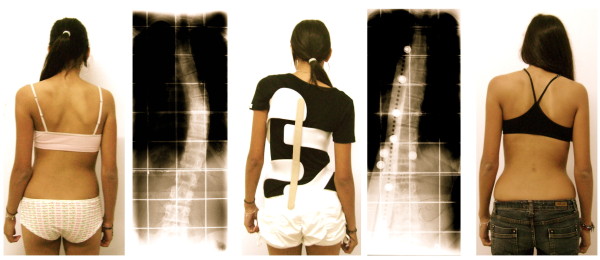
**Patient with overcorrection from 41° to - 12 after 6 weeks and clinical improvement at that stage already (*right*)**.

**Figure 15:**
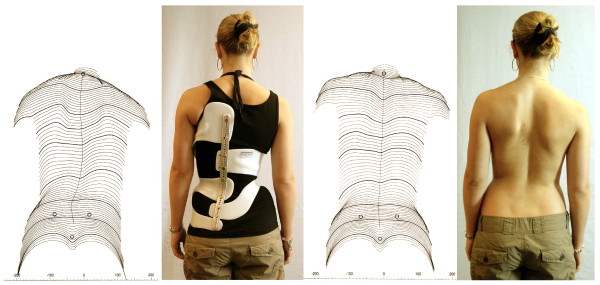
**Mature patient with good clinical correction**. This brace has been adjusted for cosmetic reasons in a mature patient at the age of 15. No significant correction of the Cobb angle has been achieved, however a significant improvement of clinical appearance, as can be seen comparing the surface scans at the start of treatment and at weaning off at the age of 17.

**Figure 16:**
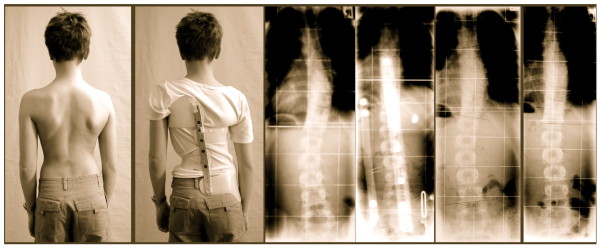
**Example of a patient with an initial overcorrection in a Chêneau light brace**. Overcorrection of a thoracic curve from 33° to -12° in a 3CL „Chêneau light" model in an 11-year old boy. The boy had been corrected to 12° without the brace on, however the boy did not comply in the end. This is why the end-result was 26°, still better than at the start of treatment.

**Figure 17:**
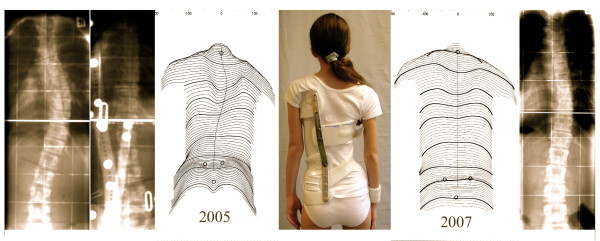
**Example of a patient with an initial overcorrection in a Chêneau light brace**. Overcorrection of a thoracic curve from 38° to -14° in a 3C „Chêneau light" model in an 11-year old premenstrual girl with Tanner II as can be seen on the *left *three pictures. After two years of treatment the curve without the brace on had been corrected to 19°.

**Figure 18:**
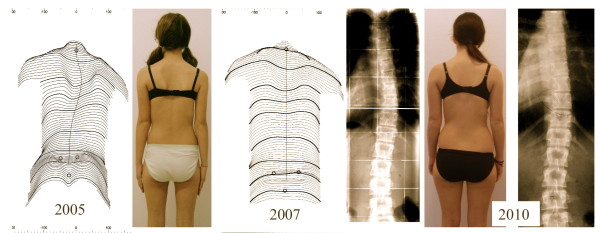
**Example of a patient with an initial overcorrection in a Chêneau light brace**. Patient from Fig. 17 with the whole documentation *left *(2005) at the start with 38°, *middle *(2007) compensated appearance with 18° and finally *right *(2010) after weaning off (at 16 years of age) with a balanced clinical appearance the curve was 12° (right).

There are, additional to the in-brace correction results and the end-results of previous Chêneau derivates, some case reports with promising intermediate and end-results, which can be demonstrated here (Fig. 13, 14, 15, 16, 17, 18).

## Discussion

Several bracing concepts are used today for the treatment of scoliosis and the in-brace corrections accepted as sufficient vary widely. The plaster cast method worldwide seems to be the most practiced technique at the moment. CAD systems are available which allow brace adjustments without plaster. The latest development however, is the ScoliOlogiC™ off the shelf system enabling the CPO to construct a light brace for scoliosis correction from a variety of pattern specific shells to be connected to an anterior and a posterior upright designed for full day treatment. The off the shelf system is named ScoliOlogiC™, while the brace after proper adjustment is called Chêneau light™ brace.

Having improved the in-brace correction of the braces also in sagittal plane, we were able to improve the correction effect in the frontal plane as well [31]. Compared to the correction effects we have achieved 2003 [8], the results now have improved significantly.

In the normal range of brace indications a correction effect of at least 20% is necessary to prevent progression [32], while a correction effect of an average 30% promises some final corrections [33]. A correction effect of 40% and more in a growing adolescent may lead to a final correction of an average 7° Cobb [6].

Wong et al. [34] have reported correction effects of an average of 40% in patients with an average Cobb degree of 30,6° (21° - 43°). However in this collective no patients with double curve patterns have been included, which generally corrected worse than single curves in our preliminary study [35].

Bullmann et al. [36] reported average correction effects of 43% in the custom Chêneau brace constructed via plaster cast in patients with a Cobb angle of 31° (25° - 40°). The final rate of success in this study however, was only 58%, which has to be regarded as rather disappointing, when compared to the success rate of 80% we reported on in another prospective study [9] with an average correction effect of less than 40% in custom Chêneau braces constructed via plaster cast (prospective controlled study) and compared to the success rate of 80% as well in another prospective study [6].

A modular 'off the shelf' orthopaedic brace for recumbent treatment has been described by Trudell [37]. This so-called "bending brace," does not correct in 3 D and the shells provided do not allow a proper adjustment for a full-day treatment. A full-day treatment, however, is necessary for a successful end result [38].

Additionally, this brace needs metal connection plates to adjust the shells to the anterior and posterior upright, whilst in the Chêneau light™ brace the shells are connected directly to the uprights giving the system the flexibility needed for the treatment of different curve patterns.

Therefore the Chêneau light™ brace can be regarded an effective tool (enabling the patient to wear full time with good correction effects) for the treatment of adolescents with scoliosis in the majority of the cases. Only certain thoracolumbar curve patterns as well as the rare left thoracic and right lumbar curves need a pattern specific CAD or plaster based construction as long as specific shells are not available to also address these curve patterns.

Increased in-brace corrections [2] and decreased in-brace stress [30] promise an effective treatment when the brace is adjusted well by a certified CPO undergoing our Quality Management (QM) procedures.

There are no end result studies on the Chêneau light™ brace, as it is a rather new application, but as it uses the Chêneau principles comparable outcomes can be assumed as in the Chêneau braces investigated previously.

There are, however some case reports with promising intermediate and end-results, which can be demonstrated within this paper (Fig. 13, 14, 15, 16, 17, 18).

## Conclusions

The use of the Chêneau light™ brace leads to correction effects above average when compared to correction effects of other braces described in literature. The reduction of material seems to increase patient's comfort and reduces the stress patients may suffer from whilst in the brace.

80% of the adolescent population of scoliosis patients can be braced with the Chêneau light™ brace. In certain patterns of curvature and in the younger population with an age of less than 11 years other approaches have to be used, such as plaster based bracing or the application of CAD/CAM based braces.

## Competing interests

The first author is applying for a patent relating to the content of this paper and is advisor of Koob-Scolitech, Abtweiler, Germany.

MW declares to have no competitive interest.

## Authors' contributions

HRW: Analysis and interpretation of data, preparation of the manuscript, acquisition of pictures and additional materials, corresponding author.

MW: Patient acquisition, pictures.

All authors read and approved the final manuscript.

## Supplementary Material

Additional file 1 **PDF file containing the basic pattern specific blueprints according to the augmented Lehnert-Schroth classification**. Click here for file

Additional file 2 **Example of a construction plan as used in Germany**. These construction plans are included with German description and serve only for documentation purposes within this article. Click here for file

Additional file 3 **Short appraisal for justification for the new brace as used in our department**. These appraisals plans are in German and serve only for documentation purposes within this article. Click here for file

Additional file 4 **CPO's checklist as used in Germany**. The checklist is in German and serves only for documentation purposes within this article. Click here for file

Additional file 5 **Physicians Checklist as used in Germany**. The checklist is in German and serves only for documentation purposes within this article. Click here for file
