# Supplementary material for: "Brace Technology" Thematic Series - The ScoliOlogiC® Chêneau light™ brace in the treatment of scoliosis
Source: Scoliosis. 2010 Sep 6;5:19. doi: 10.1186/1748-7161-5-19 (PMC2949601; doi:10.1186/1748-7161-5-19)
Supplement: Additional file 1 — PDF file containing the basic pattern specific blueprints according to the augmented Lehnert-Schroth classification. [file 1748-7161-5-19-S1.PDF]

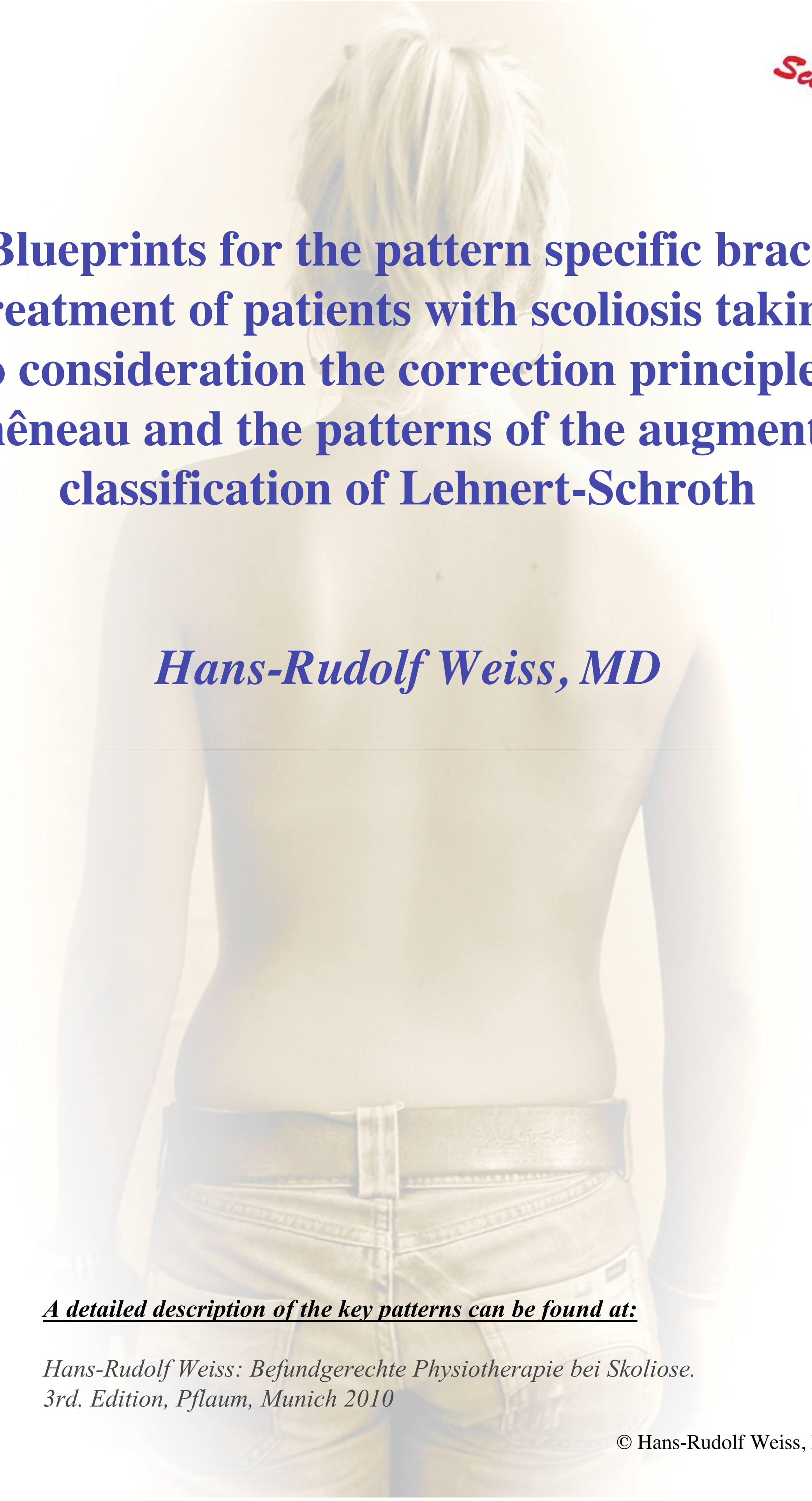

Blueprints for the pattern specific brace treatment of patients with scoliosis taking into consideration the correction principles of Chêneau and the patterns of the augmented classification of Lehnert-Schroth

Hans-Rudolf Weiss, MD

A detailed description of the key patterns can be found at:

Hans-Rudolf Weiss: Befundgerechte Physiotherapie bei Skoliose.
3rd. Edition, Pflaum, Munich 2010

© Hans-Rudolf Weiss, MD, 2010

3-curve with hip prominence (3CH)

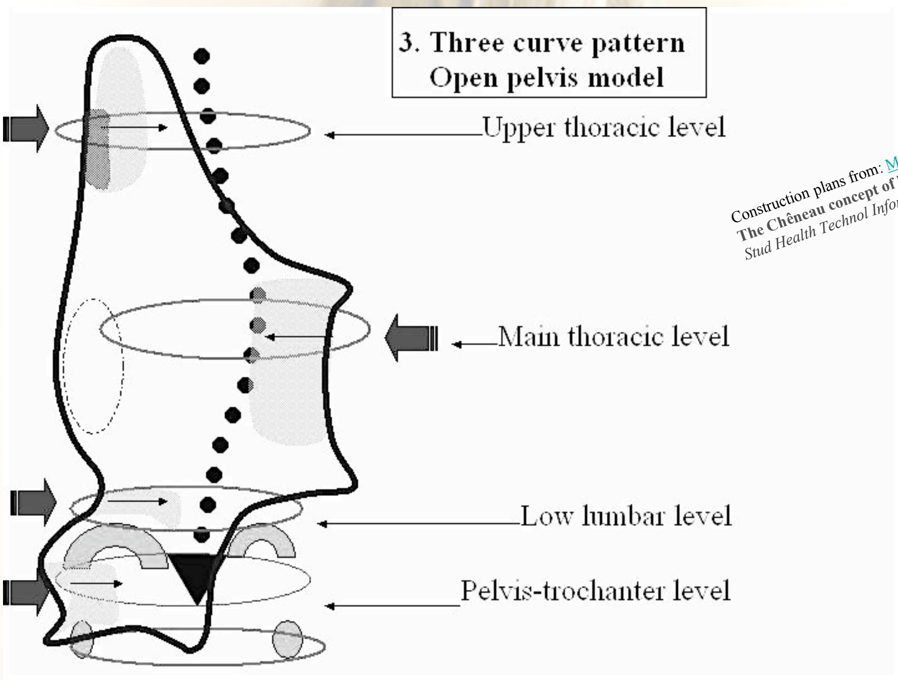

Construction plans from: Manuel Rigo, Hans-Rudolf Weiss (2008)
The Chêneau concept of bracing--biomechanical aspects.
Stud Health Technol Inform 135: 303-319

Example of treatment

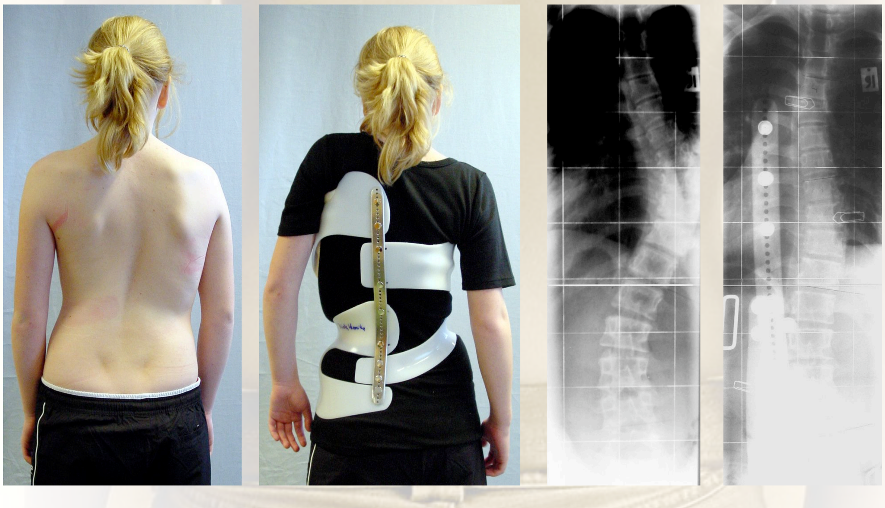

Correction from 50° to 16° in the 3CH-Chêneau light® brace. Such in-brace correction effects are only possible when the voids opposite to the corrective movement are provided.

© Hans-Rudolf Weiss, MD, 2010

3-curve balanced (3C)

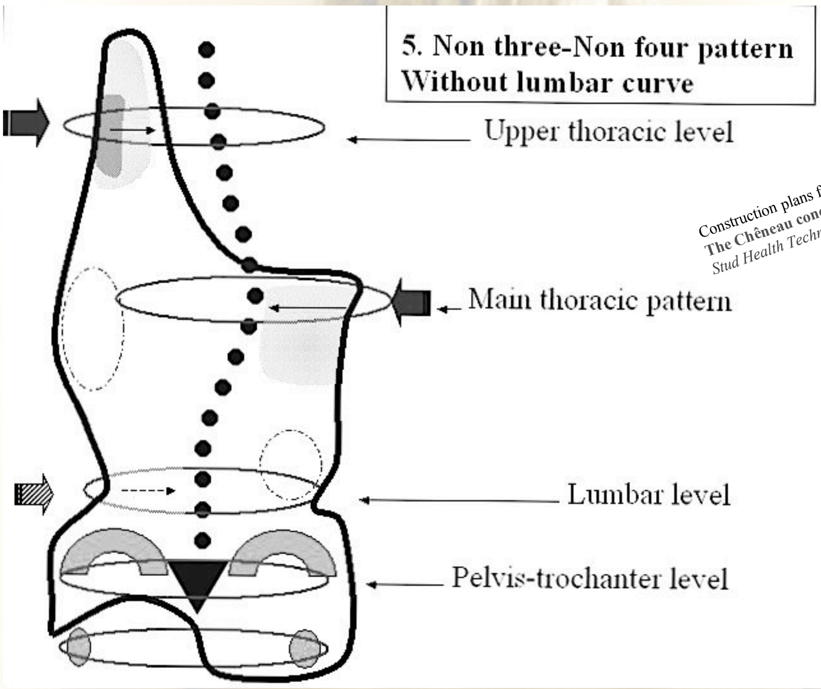

Construction plans from: Manuel Rigo, Hans-Rudolf Weiss (2008)
The Chêneau concept of bracing--biomechanical aspects.
Stud Health Technol Inform 135: 303-319

Example of treatment

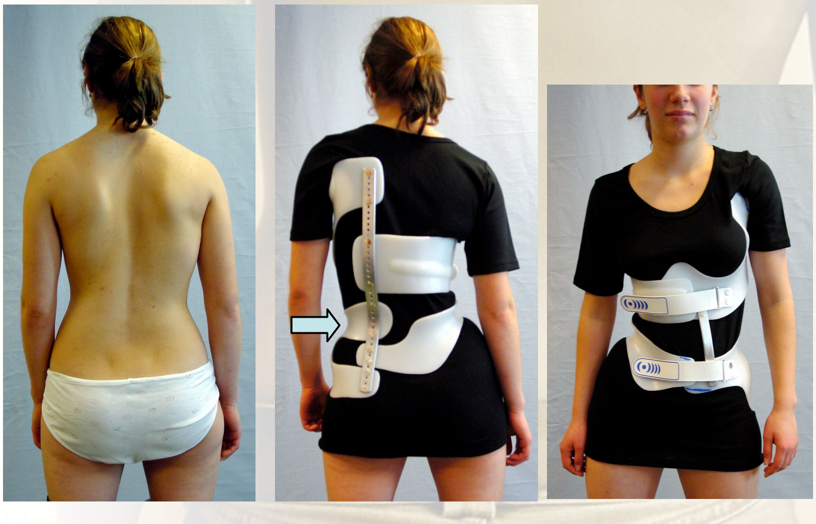

13-year old girl with 30° thoracic and 22° lumbar in the Chêneau light® brace. Mirroring of the deformity in the brace is visible. In the Chêneau light® treatment for the 3C pattern we use the functional 3-curve brace with augmentation of the lumbar pad.

© Hans-Rudolf Weiss, MD, 2010

3-curve with long lumbar curve (3CL)

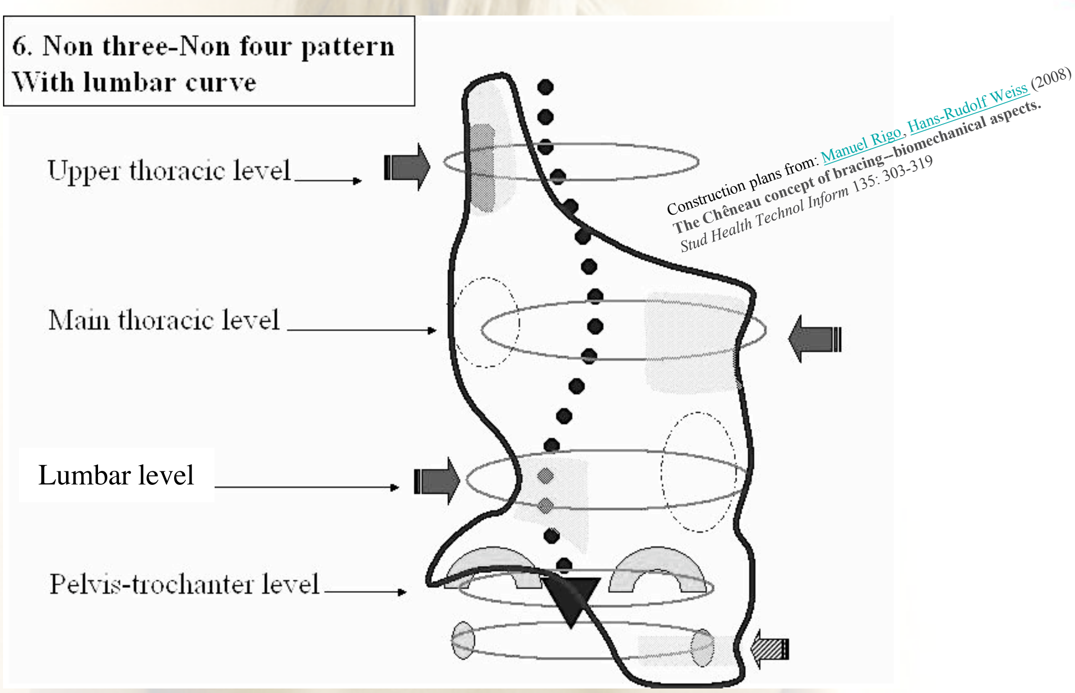

Example of treatment

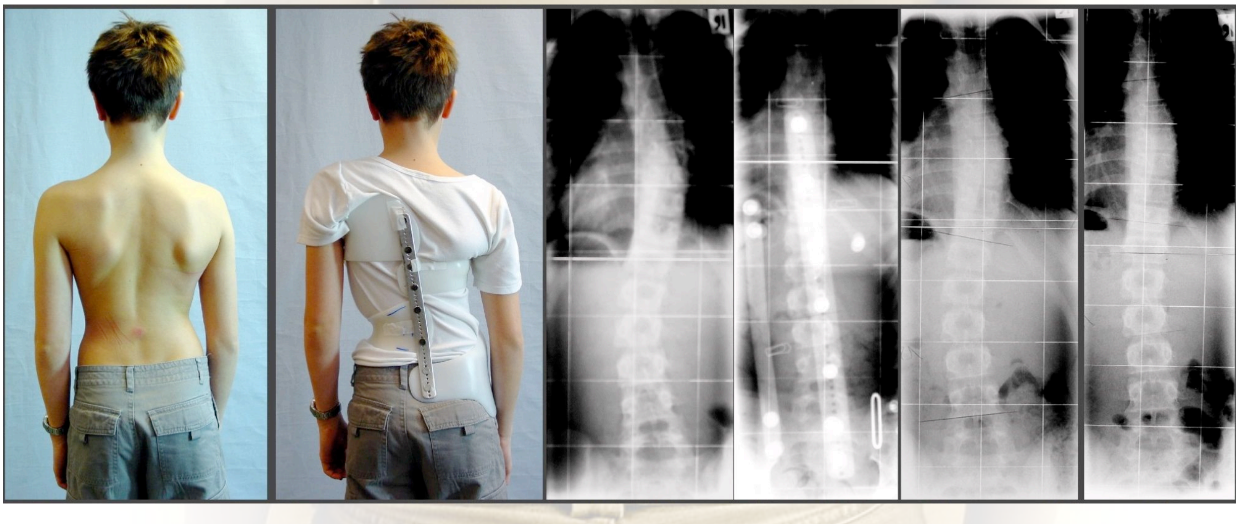

33° overcorrected to - 12°; after 7 months in the Chêneau light® brace the boy had 23° and after another 6 months 11°. From this point on part time bracing until 1 year after voice change (8-12 hrs.) was planned!
Hypercompensation to the thoracic concave side is visible, however no maximum correction of the lumbar counter curve.

© Hans-Rudolf Weiss, MD, 2010

4-curve double major (4C)

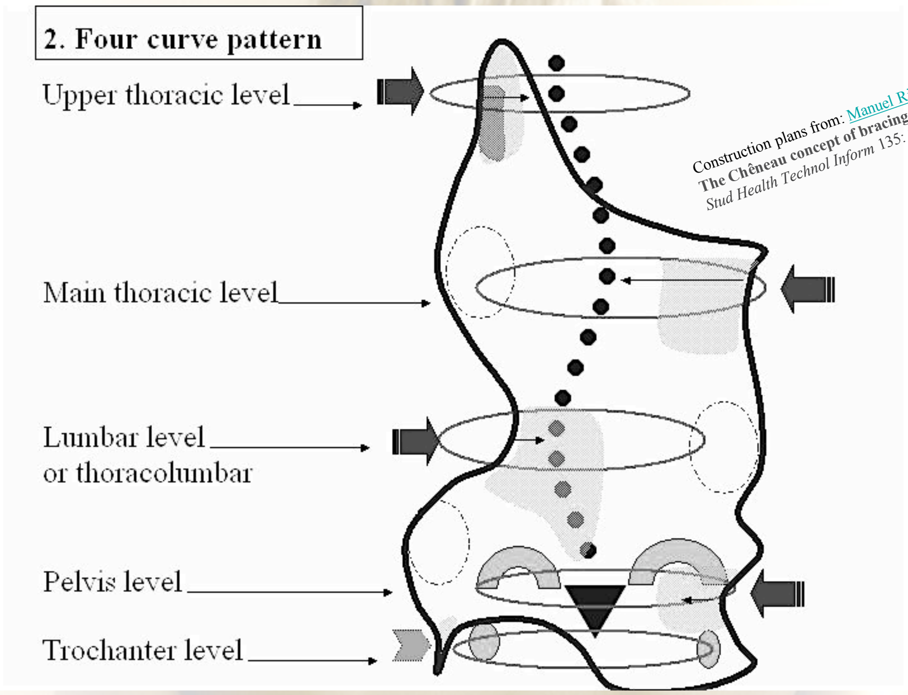

Construction plans from: Manuel Rigo, Hans-Rudolf Weiss (2008)
The Chêneau concept of bracing--biomechanical aspects.
Stud Health Technol Inform 135: 303-319

Example of treatment

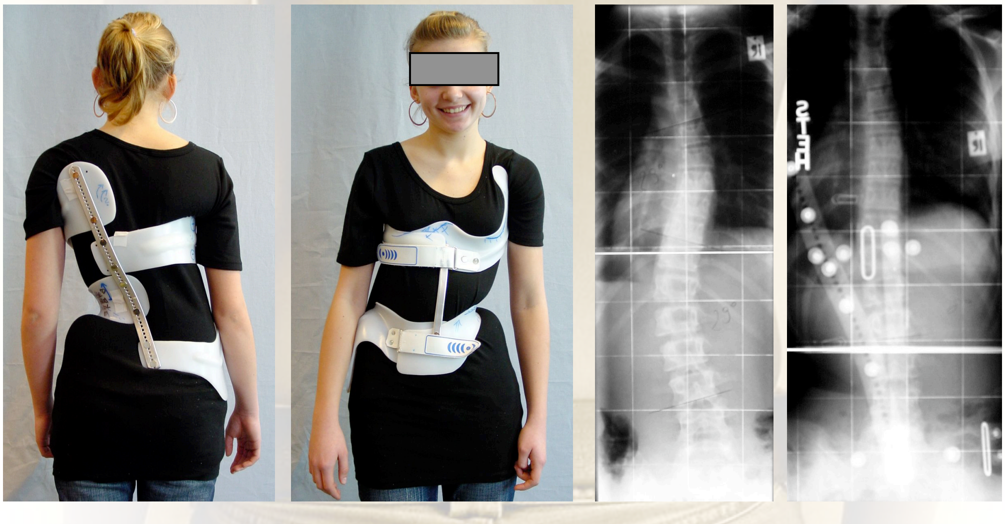

Nearly 13-year old girl with a 29 / 29° Double Major scoliosis in the ScoliOlogiC® Chêneau light® 4C-brace corrected to 11 / 9° Cobb. Mirroring of both curvatures - thoracic and lumbar as well - in the brace is clearly visible!

© Hans-Rudolf Weiss, MD, 2010

4-curve single lumbar (4CL)

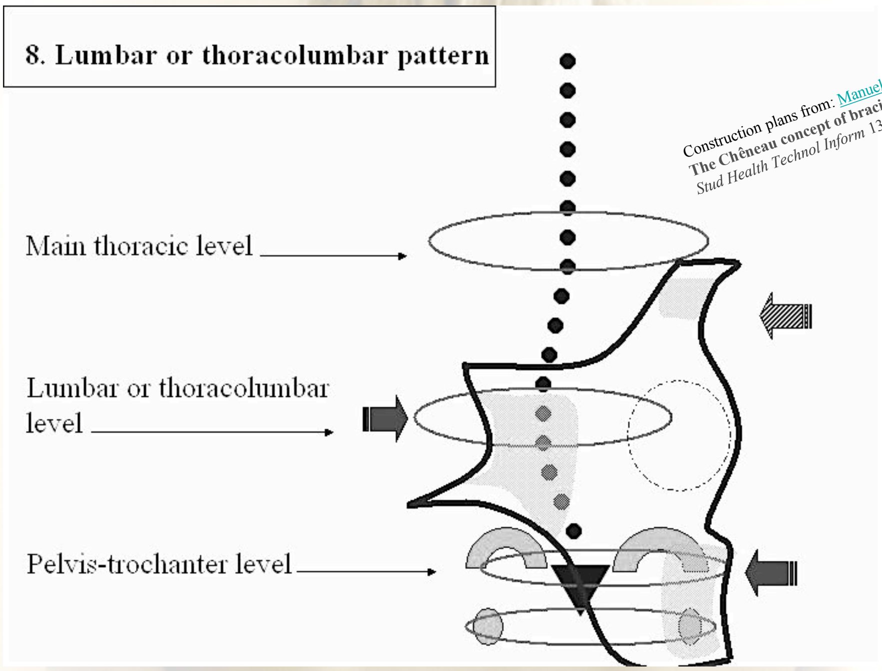

Construction plans from: Manuel Rigo, Hans-Rudolf Weiss (2008)
The Chêneau concept of bracing--biomechanical aspects.
Stud Health Technol Inform 135: 303-319

Example of treatment

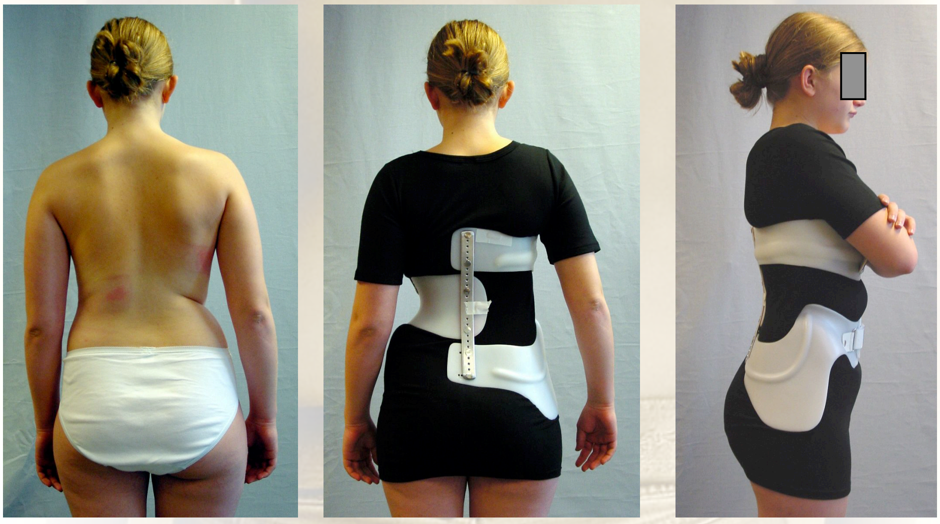

13-year old girl in the short version of the ScoliOlogiC® Chêneau light® brace. This brace mirrors the deformity and restores the sagittal profile (to be seen on the right). The Chêneau light® 4CL-brace eventually in some cases can be used for thoracolumbar curves to the left with an apical vertebra at L1.

© Hans-Rudolf Weiss, MD, 2010

3-curve thoracolumbar (3CTL)

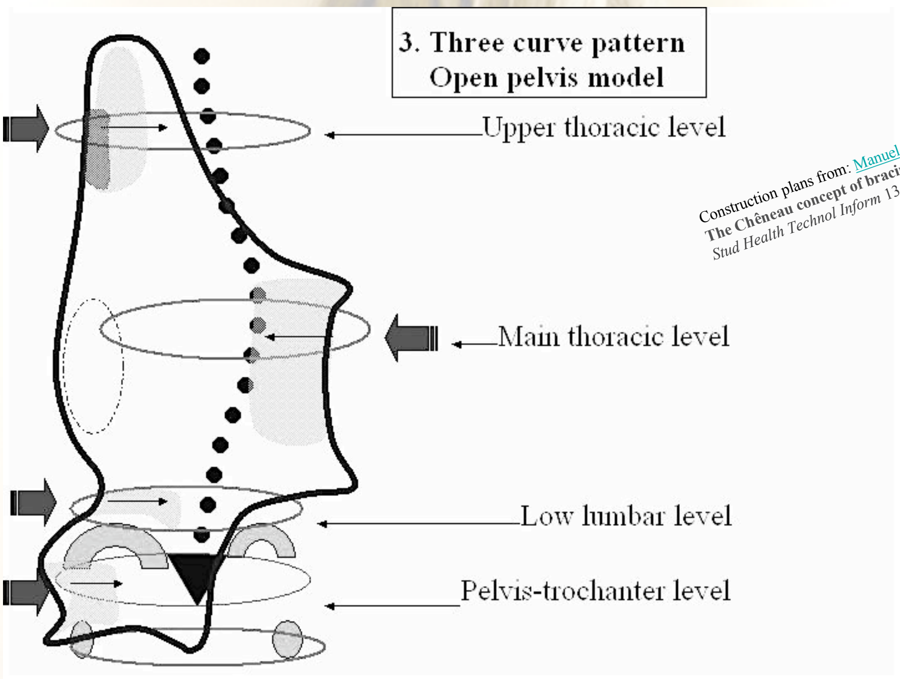

Construction plans from: Manuel Rigo, Hans-Rudolf Weiss (2008)
The Chêneau concept of bracing--biomechanical aspects.
Stud Health Technol Inform 135: 303-319

<u>Example of treatment</u>

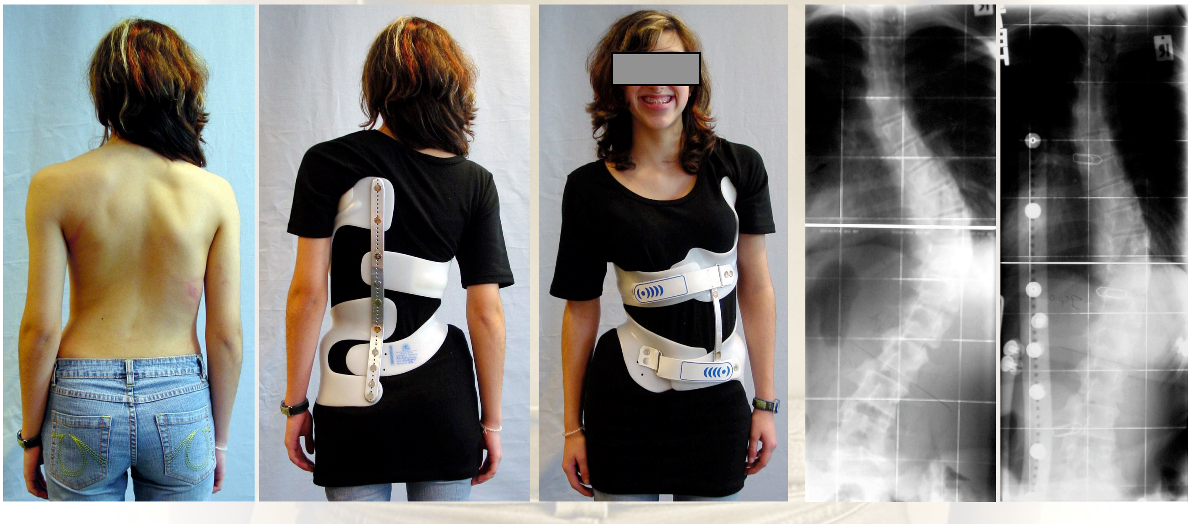

14-year old girl, Risser 2, with 62° before bracing, now in the Chêneau light® 34°. This is a variation of a 3CH treatment, with a low level thoracic shell in a thoracolumbar curve to the right with an apex at Th 12. Many of the thoracolumbar curves however, do need a custom design, because the prefabricated parts do not always fit well.

© Hans-Rudolf Weiss, MD, 2010
