# Supplementary material for: "Brace Technology" Thematic Series - The ScoliOlogiC® Chêneau light™ brace in the treatment of scoliosis
Source: Scoliosis. 2010 Sep 6;5:19. doi: 10.1186/1748-7161-5-19 (PMC2949601; doi:10.1186/1748-7161-5-19)

Arzt für Orthopädie
Physikalische & Rehabilitative Medizin
Chirotherapie, Physikalische Therapie

B Diplom der Forschungsgruppe Akupunktur
Spezialgebiet Wirbelsäulendeformitäten &

Dr. med. Hans-Rudolf Weiß
Alzeyer Str. 23
55457 Gensingen
Tel.: 06727 894040; Fax: 06727 8940429
e-mail: info@skoliose-dr-weiss.com

Dr. med. Hans-Rudolf Weiß Alzeyer Str. 23 55457 Gensingen

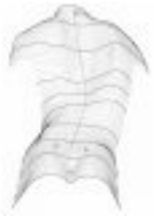

Anlage zur Verordnung einer korrigierenden Rumpforthese

Konstruktionsplan

Gensingen, den 22.07.2010

Patientin M. P., geb. am 04.02.1996, XXXXXXXXXX , XXXXXXXXXXX

Konstruktionsplan für eine Skolioseorthese entsprechend dem Schlüsselmuster T6 nach Rigo *(analog King III, non 3non 4 Korsett mit asymmetrischer Beckeneinstellung mit 4-bogiger Einstellung)*

Gesamtstatik: *Es besteht radiologisch und klinisch eine Dekompensation zur thorakalen Konvexseite, also muss das Korsett in die Gegenrichtung zur thorakalen Konkavseite hyperkompensieren.*

Beckenkorrektur: Bei schrägem Abgang der Lendenwirbelsäule auf dem Kreuzbein im Sinne einer lumbalen Krümmung ohne Keilverformung des Bandscheibenraumes L4/L5 in frontaler Ebene ist eine Beckenkippung zur thorakalen Konvexseite vorzusehen.

Lumbalkorrektur: Bei lumbalem Krümmungsscheitel bis L2 ist eine lumbale Druckzone mit Dreieckspelotte einzufügen unter Ausschluss der 11. Rippe (Blockadeeffekt mit dem thorakalen Gegenhalt und mögliche Verstärkung der thorakalen Krümmung).

Thorakalkorrektur: Bei mehrbogigen Krümmungen (double major oder doppelthorakalen Skoliosemustern aber auch bei deutlicher lumbaler Gegenkrümmung) ist die Ausrichtung der thorakalen Pelotte auf 50 bis 60° zu bringen, also mit mehr lateraler Abstützung, die ventrale Druckpelotte im Bereich Punkt 4 (nach Chêneau) sollte in der frontalen Hauptebene bis leicht frontolateral ausgerichtet sein bei einer Pelottenhöhe von nicht mehr als 3 cm kaudokranial.

Axillarkorrektur: Bei thorakalen Krümmungen ohne zervikothorakalen Gegenbogen dient die Axillarpelotte der Aufspreizung der thorakalen Konkavität, um den Ansatz der thorakalen Derotationskräfte zu verbessern. Aus diesem Grunde ist axillar kein Druck auf die oberen Rippen erforderlich, die Pelotte muss jedoch soweit herangestellt werden, dass der thorakalkonkavseitige Arm sich nicht abhebelt (Kantendruck im Bereich des Gefäß-, Nervenbündels des Armes mit der Folge: Parästhesien, venöser Rückstau). Bei thorakalem Krümmungsscheitel bis Th7 muss zusätzlich die Thorakalpelotte dorsal soweit reduziert werden, dass ohne ventrale Anlage der Schultergürtel nach dorsal kippen kann über das Hypomochlion der Thorakalpelotte am unteren Rippenbuckel. (Beim thorakalen Krümmungsscheitel höher als Th7 muss die Scheitelwirbelhöhe beachtet werden, weshalb in diesen Fällen mit mangelndem Rückhalt der thorakalkonvexseitigen Schulter eine Corakoidalpelotte notwendig werden kann).

Sagittalkorrektur: Die physiologische Lordose soll wieder hergestellt werden mit einem Scheitelpunkt bei L2 (Taillenebene). Hierzu muss im Beckenbereich dorsal ausreichend Raum vorhanden sein, damit das Becken als Gegenbewegung nach dorsal ausweichen kann. Oberhalb von L2 beginnt wieder eine Auftragungszone, so dass das Korsett von der Seite her gesehen die Form eines harmonischen „S“ aufweist.

Die Feinabstimmung bezüglich der thorakalen Segmenteinstellung und der Korrektur der kranialen (zervikothorakalen) Gegenkrümmung müssen nach Röntgenkontrolle erfolgen.

Mit freundlichen Grüßen

Dr. med. H.-R. Weiß

* Der unten gezeigte Konstruktionsplan ist eine Schemazeichnung und stellt nicht notwendiger Weise die richtigen Seitenverhältnisse dar. Bezüglich der Seitenrichtigkeit der Konstruktion beachten Sie bitte die topografischen Angaben im Text.

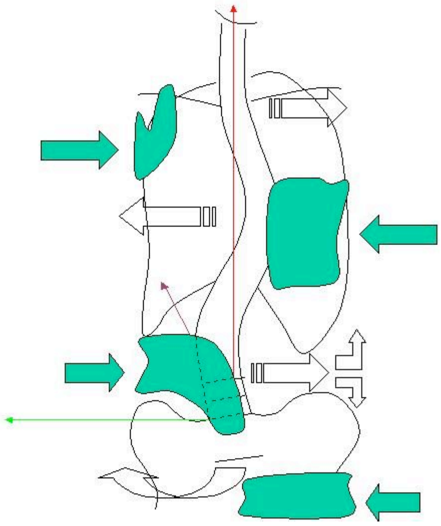

Supplement: Additional file 2 — Example of a construction plan as used in Germany. These construction plans are included with German description and serve only for documentation purposes within this article. [file 1748-7161-5-19-S2.PDF]
