# Supplementary material for: "Brace Technology" Thematic Series - The ScoliOlogiC® Chêneau light™ brace in the treatment of scoliosis
Source: Scoliosis. 2010 Sep 6;5:19. doi: 10.1186/1748-7161-5-19 (PMC2949601; doi:10.1186/1748-7161-5-19)
Supplement: Additional file 3 — Short appraisal for justification for the new brace as used in our department. These appraisals plans are in German and serve only for documentation purposes within this article. [file 1748-7161-5-19-S3.PDF]

Arzt für Orthopädie
Physikalische & Rehabilitative Medizin
Chirotherapie, Physikalische Therapie

B Diplom der Forschungsgruppe Akupunktur
Spezialgebiet Wirbelsäulendeformitäten &
[illegible]

Dr. med. Hans-Rudolf Weiß
Alzeyer Str. 23
55457 Gensingen
Tel.: 06727 894040; Fax: 06727 8940429
e-mail: info@skoliose-dr-weiss.com

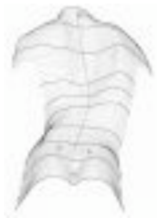

Dr. med. Hans-Rudolf Weiß Alzeyer Str. 23 55457 Gensingen

Begründung zur Veranlassung einer Neukonstruktion einer korrigierenden Rumpforthese im Wachstumsalter für

Gensingen, den 22.07.2010

Patientin M. P., geb. am 04.02.1996, XXXXXXXXX, XXXXXXXXX

Bei der heutigen Vorstellung zeigt sich, dass das Korsett aufgrund der zwischenzeitlich erfolgten Längenzunahme zu kurz geworden ist.
Die Thorakalpelotte liegt ca. 1 Querfinger unterhalb des Krümmungsscheitels und bewirkt daher wie bei einem Hypomochlion, dass sich der thorakale Krümmungsscheitel über die Pelotte lehnen kann. Somit wirkt das Korsett nunmehr die Krümmung verstärkend.
Die Axillarpelotte ist ebenfalls mittlerweile ca. 2 Querfinger zu tief und drückt etwas in die thorakale Hauptkrümmung hinein. Diese Längenveränderungen wären an sich technisch noch nachzupassen.
Es ist jedoch eine wesentliche Volumenzunahme erfolgt, da die Freiräume nicht mehr offen sind. Der Freiraum zwischen Punkt 3 und Punkt 4 nach Chêneau ist mit dem Finger nicht mehr unterfahrbar, ebenso wenig wie der Freiraum ventral des Rippenbuckels. Es ist daher eine bedeutsame Beeinträchtigung der Atemfunktion eingetreten, das der Rippenkorb nicht mehr ausreichend expandieren kann. Ferner stehen keine Freiräume zur Volumenverschiebung mehr zur Verfügung, so dass keine Korrektur mehr möglich ist.

An den Beckenkämmen zeichnen sich zudem mittlerweile die Passteile des Korsetts deutlich als Druckstellen ab.

Aufgrund der mittlerweile zu weit vom Krümmungsscheitel entfernt liegenden Hauptdruckzonen in Thorakalbereich und aufgrund des bestehenden Volumenmangels durch Zunahme des knöchernen Breitenwachstums können Nachbesserungen leider nicht mehr erfolgen.

Es ist daher dringend Neuversorgung innerhalb der nächsten 14 Tage erforderlich, damit nicht zwischenzeitlich eine Verschlechterung eintritt.

Mit freundlichen Grüßen

Dr. med. H.-R. Weiß
