# Supplementary material for: "Brace Technology" Thematic Series - The ScoliOlogiC® Chêneau light™ brace in the treatment of scoliosis
Source: Scoliosis. 2010 Sep 6;5:19. doi: 10.1186/1748-7161-5-19 (PMC2949601; doi:10.1186/1748-7161-5-19)
Supplement: Additional file 4 — CPO's checklist as used in Germany. The checklist is in German and serves only for documentation purposes within this article. [file 1748-7161-5-19-S4.PDF]

Checkliste vor erster Korsettvorstellung beim Arzt durchgehen!
(Chêneau light®)

Unmittelbar vor Abgabe und vor der ärztlichen Kontrolle sollte die im Folgenden beschriebene Checkliste abgearbeitet werden. Erst nach endgültigem Beschnitt und erst kurz vor Vollendung des Korsetts sollen die Verschlüsse angebracht werden, um Verwringungen zu vermeiden und ein Umschlagen der Umlenkrollen zu verhindern.

Lumbalkrümmungen:

- Spiegelt das Korsett das Krümmungsmuster? √
- Biegt sich die Hauptkrümmung im Scheitel in die Gegenrichtung? √
- Steht der Beckenkamm auf der Seite von Formteil B tiefer als auf der Seite von Formteil L? √
- Ist das Formteil B kaudal mehr medialwärts gekippt als kranial? √
- Kann ich Formteil L mit dem Finger oberhalb des Beckenkamms vollständig unterfahren? √
- Stört Formteil L die Thorakalkorrektur? (11. Rippe seitlich) √
- Sind die Längsträger ventral und dorsal ordnungsgemäß geschränkt? √

Thorakalkrümmungen:

- Spiegelt das Korsett das Krümmungsmuster? √
- Biegt sich die Hauptkrümmung im Scheitel in die Gegenrichtung? √
- Kann ich Formteil BR mit dem Finger oberhalb des √
- Beckenkamms vollständig unterfahren? √
- Stört Formteil L die Thorakalkorrektur? (11. Rippe seitlich) √
- Überragt oder unterfährt Formteil Th seitlich die Scheitelwirbelhöhe? √
- Lässt Formteil Th den Bereich zwischen ventraler Axillarlinie auf Seiten der Thorakalkrümmung und der gegenseitigen Parasternallinie bei maximalem Verschluss des Korsetts frei? √
- Ist der Raum zwischen Punkt 3 und Punkt 4 des Formteils Ax bei maximalem Verschluss des Korsetts frei? √
- Ist der dorsolaterale Winkel von Formteil Th ausgepolstert mit Gegenpelotte bei Punkt 4? √
- Kann Formteil Ax noch weiter nach kranial verschoben werden? √
- Ist ein Kantendruck in Druckzone 3 ausgeschlossen? √
- Sind die Längsträger ventral und dorsal ordnungsgemäß geschränkt? √

Ausdrücklich sind hier keine speziellen Krümmungsmuster (King V o. Ä.) berücksichtigt. Die Checkliste spiegelt die Grundeinstellung des Chêneau light® Korsetts wieder für thorakale und lumbale Krümmungen. Sie kann alerdings auf alle Chêneau modifikationen übertragen werden.
